# A deep learning-based method for drug-target interaction prediction based on long short-term memory neural network

**DOI:** 10.1186/s12911-020-1052-0

**Published:** 2020-03-18

**Authors:** Yan-Bin Wang, Zhu-Hong You, Shan Yang, Hai-Cheng Yi, Zhan-Heng Chen, Kai Zheng

**Affiliations:** 1 Xinjiang Technical Institute of Physics and Chemistry, Chinese Academy of Sciences, Urumqi, 830011 China; 2 Department of Computer Science and Technology, University of Chinese Academy of Sciences, Beijing, 100049 China

**Keywords:** Drug-target, Deep learning, Legendre moment, Long short-term memory

## Abstract

**Background:**

The key to modern drug discovery is to find, identify and prepare drug molecular targets. However, due to the influence of throughput, precision and cost, traditional experimental methods are difficult to be widely used to infer these potential Drug-Target Interactions (DTIs). Therefore, it is urgent to develop effective computational methods to validate the interaction between drugs and target.

**Methods:**

We developed a deep learning-based model for DTIs prediction. The proteins evolutionary features are extracted via Position Specific Scoring Matrix (PSSM) and Legendre Moment (LM) and associated with drugs molecular substructure fingerprints to form feature vectors of drug-target pairs. Then we utilized the Sparse Principal Component Analysis (SPCA) to compress the features of drugs and proteins into a uniform vector space. Lastly, the deep long short-term memory (DeepLSTM) was constructed for carrying out prediction.

**Results:**

A significant improvement in DTIs prediction performance can be observed on experimental results, with AUC of 0.9951, 0.9705, 0.9951, 0.9206, respectively, on four classes important drug-target datasets. Further experiments preliminary proves that the proposed characterization scheme has great advantage on feature expression and recognition. We also have shown that the proposed method can work well with small dataset.

**Conclusion:**

The results demonstration that the proposed approach has a great advantage over state-of-the-art drug-target predictor. To the best of our knowledge, this study first tests the potential of deep learning method with memory and Turing completeness in DTIs prediction.

## Background

Drug targets are the foundation of drug research and development, and over the past few centuries, people have relied heavily on hundreds of drug targets currently known to detect drugs [1]. Although the number of known drugs interacting with target proteins continues to increase, the number of approved drug targets is still only a small fraction of the human proteome. The detection of interactions between drugs and targets is the first step in the development of new drugs, and one of the key factors for drug screening and drug directed synthesis. Benefit from high-throughput experiments, more and more understanding of the structural space of drug compounds and the genomic space of target proteins has been made. Unfortunately, due to the time-consuming and laborious experimental process, our understanding of the relationship between the two spaces is still rather limited [2, 3]. Thanks to the rapid increase in publicly available biological and chemical data, researchers can systematically learn and analyze heterogeneous new data through computational methods and revisit drug-target interactions (DTIs). There are several free databases that focus on relationships between drugs and targets, such as the ChEMBL [4], DrugBank [5], SuperTarget [6]. These database contents constitute the gold standard datasets, which are essential for the development of computational methods to predict DTIs.

At present, the computational method for DTIs prediction can be classified into three categories: the ligand-based approach, the docking approach and the feature learning approach. Ligand-based methods are often used to estimate potential targets of action by calculating the chemical structural similarity of a given drug or compound to active compounds of known targets. Keiser et al. [3] proposed a method for inferring protein targets based on the chemical similarity of their ligands. Yamanishi et al. [7–9] predict unknown drug-target interactions by integrating the chemical structural similarity of compounds and the amino acid sequence similarity of proteins to a uniform space. Campillos et al. [6] predict the potential target proteins through similarity of phenotypic side effects. This kind of ligand-based method is simple and effective in the case of high chemical structural similarity, but it also limits the scope and accuracy of its application to a great extent. The docking method is to calculate the shape and electrical matching of drugs and potential targets in three-dimensional structure, thereby inferring possible targets of action of the drug. Among them, the reverse docking method is the most commonly used prediction method. This method ranks drug targets by predicting the interaction mode and affinity between a given compound and a target, thereby determining possible targets for the drug. Cheng et al. [10] developed a structure-based maximum affinity model. Li et al. [11] developed a web server called TarFisDock that uses docking methods to identify drug targets. Such methods fully consider the three-dimensional structural information of the target protein, but the molecular docking method itself still has some problems that have not yet been effectively solved, such as protein flexibility, the accuracy of scoring functions, and solvent water molecules, which lead to reverse docking. The prediction accuracy of the method is low. Another serious problem with docking is that it cannot be applied to proteins with unknown 3D structures. So far, proteins with known 3D structure are still only a small part of all proteins. This severely limits the promotion and popularization of this method. A feature learning approach treats drug target relationships as a two-class problem: interaction and non-interaction. Such methods learn the potential patterns of known compound-target pairs using machine learning algorithms, generate prediction models by iterative optimization, and then infer potential DTIs. Yu et al. [12] proposed a systematic approach based on chemical, genomic, and pharmacological information. Faulon et al. [13] predicted drug targets using the signature molecular descriptor. Even though these methods have accelerated the discovery of drug targets, there is still much room for improvement.

**Fig. 1:**
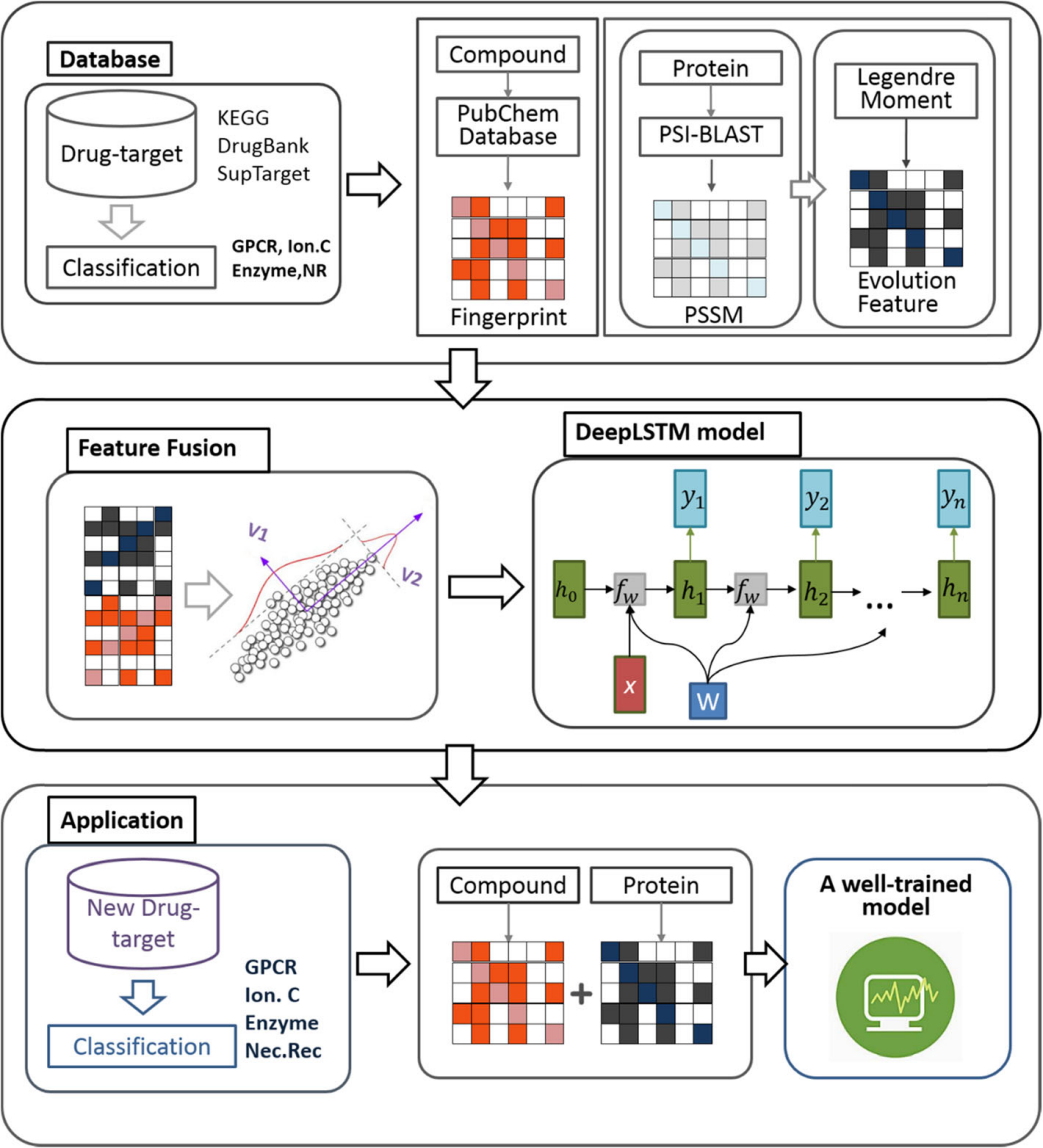
Schematic diagram of drug targets predicted by the proposed method

In this work, we proposed deep learning-based method to identify unknown DTIs. The proposed method consists of three steps: (i) Representation for drug-target pairs. The drug molecules are encoded as fingerprint feature and the protein sequences features are obtaining by using Legendre Moments (LMs) on Position Specific Scoring Matrix (PSSM) that contains evolutionary information about protein. (ii) Feature compression and fusion. The Sparse Principal Component Analysis (SPCA) is used to decrease the features dimension and information redundancy. (iii) Prediction. The Deep Long Short-Term Memory (DeepLSTM) model is adopted for executing prediction tasks. The flow of our proposed model is represented in Fig. 1. We implement the proposed method on four important DTIs datasets involving *enzymes*, *ion channels*, *GPCRs* and *nuclear receptors*. The results are exposed to give superior performance to the existing state-of-the-art algorithms for DTI prediction.

## Materials and methods

### Data collection

We collected information about the interactions between drug compounds and target proteins form KEGG [14], DrugBank [5], and SuperTarget [6] databases [14, 15]. Table 1 summarizes the data set according to the number of drug compounds, and target protein and interactions. This set of known DTIs are considered to be the gold standard for assessing the performance of the proposed method. Target proteins are linked to drug molecules to form a network of drug targets. To obtain positive datasets from the network, all identified drug-target pairs in gold standard dataset are considered as positive samples. The negative sample correspond to the remaining drug-target pairs in the network. Since the scale of the non-interaction pairs is much larger than that of the interaction pairs, the constructed datasets are imbalanced. In order to solve the bias caused by imbalanced data sets, we randomly selected negative samples from the remaining drug-target pairs in the network, until the number of negative samples is the same as that of positive samples.

**Table 1:** The selected drug-target interaction data sets from KEGG, SuperTarget, and DrugBank databases

Dataset	Interactions	Targets	Drugs
Enzyme	2926	664	445
Ion channel	1476	204	210
GPCR	635	95	223
Nuclear receptor	90	26	54

### Characterization of drug molecules

The ability of substructure fingerprints in characterizing drug molecules has been confirmed in some studies. Through the comprehensive analysis of previous research results, PubChem fingerprint was used to characterized each drug molecules. In this work, drugs are encoded Boolean substructure vector representing the presence or absence of corresponding substructures in a molecule. The PubChem database defines 881 chemical substructures in which each substructure is assigned to a particular location. Therefore, for a substructure appears in the drug compound, the position corresponding to the substructure in the fingerprint vector is set to 1, otherwise, and the corresponding position is set to 0. Hence, each drug was represented as an 881-dimensional vector [16].

### Characterization of target proteins

#### Position specific scoring matrix

The position specific scoring matrix (PSSM) was firstly introduced for finding distantly related proteins. In recent years, PSSMs is widely used in proteomics and genomics research, such as prediction of DNA or RNA binding sites and membrane protein types. In this paper, PSSM is used to encode proteins and obtain evolutionary information about amino acids. The PSSM of protein *A* with *N* amino acids residue can be expressed as

1 $$ {A}_{PSSM}=\left[\begin{array}{cccccc}{A}_{1\to 1}& {A}_{1\to 2}& \dots & {A}_{1\to j}& \dots & {A}_{1\to 20}\\ {}{A}_{2\to 1}& {A}_{2\to 2}& \dots & {A}_{2\to j}& \dots & {A}_{2\to 20}\\ {}\vdots & \vdots & \vdots & \vdots & \vdots & \vdots \\ {}{A}_{i\to 1}& {A}_{i\to 2}& \dots & {A}_{i\to j}& \dots & {A}_{i\to 20}\\ {}\vdots & \vdots & \vdots & \vdots & \vdots & \vdots \\ {}{A}_{N\to 1}& {A}_{N\to 2}& \dots & {A}_{N\to j}& \dots & {A}_{N\to 20}\end{array}\right] $$

where *A*_*i* → *j*_ is a score that represents probability of *i-th* residue being mutated to *j-th* native amino acid and *N* is the length of amino acids residue of sequence *A,* 20 means the 20 native amino acid types. To get the PSSM for each protein sequence, the Position Specific Iterated BLAST (PSI-BLAST) [17, 18] was utilized and the default parameters were choosing except for three iterations [19, 20].

#### Legendre moments

The invariant moments are a global statistical feature and has excellent characteristics in size invariance, rotation invariance and displacement invariance which avail to the extraction of stability features. Legendre moments (LMs), as a fast moment invariant feature extraction technology, show good performance in the application of many pattern recognition, viz., graphic analysis, target recognition, image processing, classification and prediction. Here, we use Legendre moment to further refine the evolutionary information contained in PSSM and generate feature vector. LMs are continuous orthogonal moments, which can be used to represent objects with minimal information redundancy [21, 22]. The LMs with order (*a*, *b*) are defined as

2 $$ {L}_{ab}=\frac{\left(2a+1\right)\left(2b+1\right)}{4}\sum \limits_{i=1}^C\sum \limits_{j=1}^V{h}_{ab}\left(x,y\right)I\left({x}_i,{y}_i\right) $$

where *I*(*x*, *y*) is a set of discrete points (*x*_*i*_, *y*_*i*_), *x*_*i*_, *y*_*i*_ ∈ [−1, +1]. In this work, *I*(*x*, *y*) denotes PSSM, C is the number of rows of a PSSM, V means the sum of each column of a PSSM [23, 24]. The

3 $$ {h}_{ab}\left(x,y\right)={\int}_{x_i-\frac{\Delta  x}{2}}^{x_i+\frac{\Delta  x}{2}}{\int}_{y_i-\frac{\Delta  y}{2}}^{y_i+\frac{\Delta  y}{2}}{R}_a(x)\ {R}_b(y) dxdy $$

where

4 $$ {R}_a(x)=\frac{1}{2^aa!}\frac{d^a}{dx^a}{\left({x}^2-1\right)}^a=\frac{1}{2^a}\sum \limits_{k=0}^{\left[a/2\right]}-{1}^k\left(\genfrac{}{}{0pt}{}{p}{k}\right)\left(\genfrac{}{}{0pt}{}{2\left(p-k\right)}{p}\right){x}^{p-2k} $$

The integral terms in (3) are commonly estimated by zeroth-order approximation, that is, the values of Legendre polynomials are always to be constant over the intervals [${x}_i-\frac{\Delta  x}{2},{x}_i+\frac{\Delta  x}{2}$] and [${y}_i-\frac{\Delta  x}{2},{y}_i+\frac{\Delta  x}{2}$]. Hence, the set of approximated LMs is defined as:

5 $$ L{`}_{ab}=\frac{\left(2a+1\right)\left(2b+1\right)}{KL}\sum \limits_{i=1}^K\sum \limits_{j=1}^L{R}_a\left({x}_i\right)\ {R}_b\left({y}_i\right)g\left({x}_i,{y}_i\right) $$

As a result, using LMs on PSSM of protein sequence, we have obtained 961 features from each protein sequence by setting *a*, *b* = 30.

### Feature compression and fusion

We got an 1842-dimensional drug target feature vector from each drug target pair by combining drug substructure fingerprint features (881-D) with protein LMs features (961-D). To economize calculating time of classifier, reduce memory consumption and remove noisy features from the original feature space, the sparse principal component analysis (SPCA) is used to integrate both features of drugs and target proteins into an organic whole, reduce the feature dimension and redundant information. Classical principal component analysis (PCA) has an obviously drawback, that is, each PC is a linear combination of all variables and the loadings are typically nonzero. Thus, when dealing with a combination of two different types of features, such as the drug and protein features produced herein, often results in unpredictable results. SPCA is an improved PCA the using lasso (elastic net) to produce principal components with sparse loadings, which overcome above problem. Finally, we gain 400-dimensional refined feature vector as the input of classifier.

## Constructing DeepLSTM model

LSTM is a special recurrent neural network (RNN) architecture, providing more excellent performance than the traditional RNNs [25]. In this section, we explore the application of LSTM architecture in predicting drug-target.

**Fig. 2:**
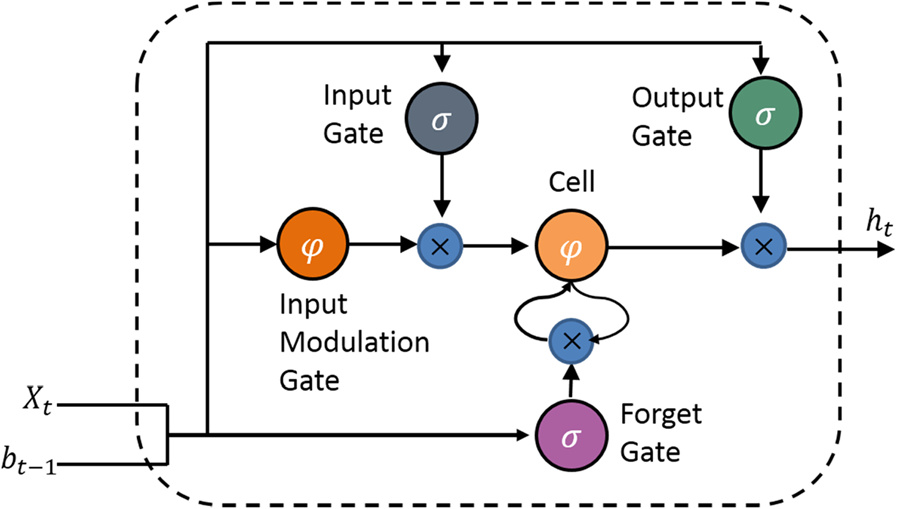
Memory block of LSTM networks

One of the major differences with standard RNNs network is the LSTM architecture use memory blocks to replace the summation units. Memory blocks, as shown in Fig. 2, contain self-connection memory cells for storing the temporal state, and gates (special multiplicative units), input gate, output gate and forget gate, for controlling the information flow. To better understand the work of the gate unit, memory cells are not shown in the Fig. 2. These gates enable the LSTM to store and access over lengthy periods of time, thereby reducing the impact of vanishing gradient problems on the prediction model. The input activation flow that enters the memory unit is controlled by the input gate [26, 27]. The output flow of cell activation flows to other parts of the network, which is dominated by the output gate. Through the self-recursive connection of the unit, the forgetting gate is added to the cell as input, so that the LSTM network can process the continuous input stream. In addition, the LSTM cell can include peephole connections, that allow gates to be modulated according to the state values in the internal memory [28].

We constructed DeepLSTM by stacking multiple LSTM layers [29, 30]. Compared with simple three-tier architecture, deep architecture can better use the parameters through the distribution of multiple layers in space. Deep results in inputs going through more non-linear operations per time step.

### Prevent over fitting

**Fig. 3:**
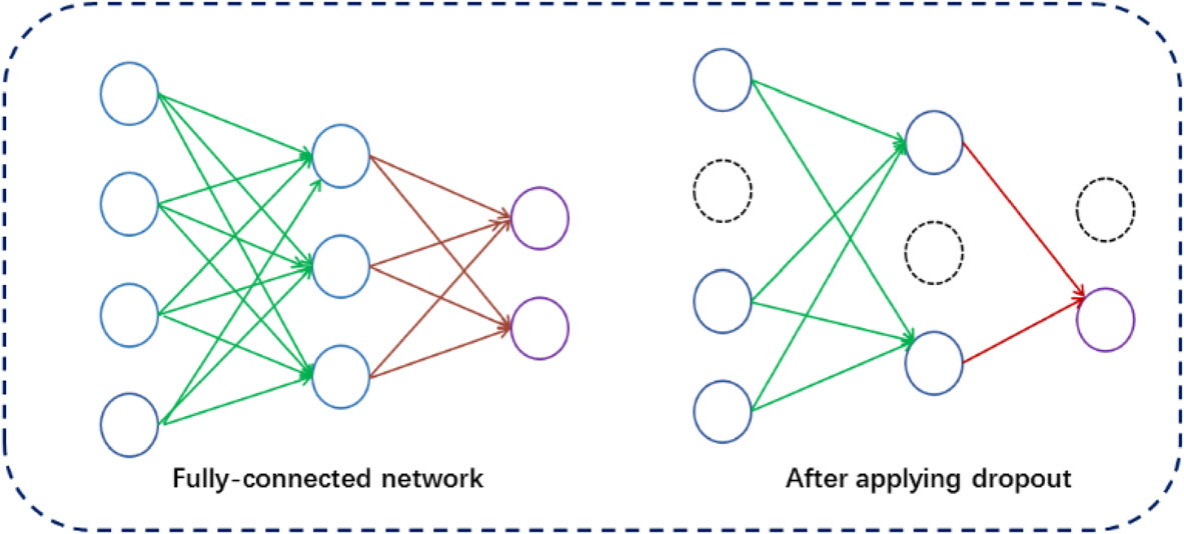
Dropout Neural Net Model. Left: A standard full connection network; Right: A thinned network generated by utilizing dropout in Left

Neural networks often optimized with a large number of parameters. However, there may be overfitting problems in such networks. Dropout is used for solving this problem by randomly removing units from the neural network and their connections in the train of training. The meaning of “dropout” is to extract a “sparse” network from the original network, the sparse network is composed of all the surviving units, as shown in Fig. 3. In this paper, we follow the previous study to set the dropout rate to 0.5. We have 35 hidden layer units, which may generate 2^35^ different subnets during training. In the testing phase, an “mean network” strategy is adopted, which contains all of the original network connection, but their efferent weights are halved in order to make up for the fact that twice as many of them are active [31, 32].

## Experiment settings

### Evaluation indicators

In this paper, we evaluate the performance of our predictor by calculating accuracy (ACC), true positive rate (TPR), specificity (SPC), positive predictive value (PPV), and Matthews’s correlation coefficient (MCC). The ACC is used to reveal the overall level of prediction. The TPR exposes the proportion of positives samples that have been correctly predicted in the test results. The SPC exposes the proportion of negatives samples that have been correctly predicted in the test results. The PPV is used to reveal the proportion of the true positive samples in the samples that were predicted to be positive. The MCC is a general measure of predictive performance for two classification problems. These performance indicators are defined as follow:

6 $$ \mathrm{ACC}=\frac{TN+ TP}{TN+ FN+ TP+ FP} $$

7 $$ \mathrm{TPR}=\frac{TP}{FN+ TP} $$

8 $$ \mathrm{SPC}=\frac{TN}{TN+ FP} $$

9 $$ \mathrm{PPV}=\frac{TP}{TP+ FP} $$

10 $$ \mathrm{MCC}=\frac{\left( TP\times TN\right)+\left( FP\times FN\right)}{\sqrt{\left( TP+ FP\right)\times \left( TN+ FN\right)\times \left( TP+ FN\right)\times \left( TN+ FP\right)}} $$

Here, *FN*, *FP*, *TN*, *TP* represents the number of false negative, false positive, true negative and true positive, respectively, and the area under the Receiver Operating Characteristic curve (AUC) is calculated used for measuring the quality of prediction [33–35].

### Model training

For four datasets, we divided each dataset into: the training set; the verification set; the test set. Test sets account for one tenth of the total, the training set account for eight tenths of the remaining data, the rest are used as validation sets. We use the training set to fit a DeepLSTM prediction model, use the validation set to optimize the DeepLSTM neural network weight, use the test set to verify the model performance. Another benefit of using validation set is to prevent overfitting by early stopping: terminate model training when errors on the validation dataset no longer decrease and have an increasing trend. This trick avoids the overfitting and reduces the training cost of the model.

We use hyperbolic tangent activation for the cell input units and cell output units, and logistic sigmoid for the input, output and forget gate units. The input to the LSTMs and RNNs is 40-dimensional features. The output layer is a fully connected network and uses softmax function to produce probability results. In order to find the best network structure, we test the performance of DeepLSTM models with different number of layers and units on the validation data. The number of hidden layers that were trialed from 1 to 6. With respect to the number of units, these were trialed from 20 to 200 with stride s = 4. Finally, the DeepLSTM model with 4 hidden layers and 36 units was determined. The weights of the DeepLSTM were initialized using random numbers with 0 mean and standard deviation 0.1. We trained model with mean squared error and *Nadam* optimizer, using dynamic learning rate with initial value of 0.002, decay of 0.004 and momentum of 0.5. The time step was set to 1 and batch size was 64. Training was stopped after a maximum of 500 iterations or early stopping if there was no new best error on the validation data.

## Results and discussion

Statistics of the prediction performance for the proposed models are given in Table 2. Focus on *enzymes* data sets, our predictor has given satisfying result of 92.92% accuracy, along with of 99.31% sensitivity, of 86.57% specificity, of 88.04% precision, of 86.75% MCC and AUC of 0.9951. The same good results also appear on other three data sets by using our method. The results achieved of our method on *ion channels* dataset is 91.97% accuracy, along with 93.23% sensitivity, of 90.87% specificity, of 89.95% precision, of 85.19% MCC and AUC of 0.9705. The results achieved of our method on *GPCRs* dataset is 91.80% accuracy, along with 83.71% sensitivity, of 100% specificity, of 100% precision, of 84.44% MCC and AUC of 0.9511. The results achieved of our method on *nuclear receptors* dataset is 91.11% accuracy, along with 95.24% sensitivity, of 87.50% specificity, of 86.96% precision, of 83.76% MCC and AUC of 0.9206. There is particularly noteworthy is our method achieved over 90% accuracy on nuclear receptors datasets with only 180 sample. This clearly shows that our method can provide excellent performance in the case of very small training samples. This is a huge advantage that will be clearly distinguished from other methods. The extraordinary performance comes mainly from the following three points: 1) our feature representation method can effectively extract the discriminative features from drug molecular and target protein sequence; 2) SPCA enjoys advantages in several aspects, including computational efficiency, high explained variance and an ability in identifying important variables, which compresses two different feature vectors into a unified feature space and extracts heterogeneous features; 2) The hierarchical structure enables the neural network to convert the input data into new feature space which is more conducive to complete classification tasks.

**Table 2:** Prediction performance for the four datasets in term of ACC, TPR, SPC, PPV, precision, MCC, and AUC

Model	Data Sets	ACC (%)	TPR (%)	SPC(%)	PPV (%)	MCC (%)	AUC
DeepLSTM	*enzymes*	92.92	99.31	86.57	88.04	86.75	0.9951
	*ion chan.*	91.97	93.23	90.87	89.95	85.19	0.9705
	*GPCRs*	91.80	83.71	100	100	84.44	0.9951
	*nucl. rec.*	91.11	95.24	87.50	86.96	83.76	0.9206

### Comparison with others classifier model

To exhibit the advantage of DeepLSTM, computations were performed on *enzymes, ion channels, GPCRs and nuclear receptors* datasets by using other two prominent classifiers (Multi-layer Perceptron and Support Vector Machines). For fairness, except for the different classifiers, the other settings are completely consistent. We build multi-layer perceptron (MLP) networks, in which the number of hidden layers and neurons is the same as the DeepLSTM network. The Support Vector Machine (SVM) was available by using LIBSVM tool [36]. The parameters are optimized by grid search technology. The results of 5-fold cross-validation achieved by SVM can be found in Tables S1, S2, S3 and S4 of the Supplementary Material. The cross validation average results on four datasets are presented in the Table 3.

**Table 3:** Comparison with three classifier on four datasets in term of ACC, TPR, SPC, PPV, precision, MCC, and AUC

Datasets	Model	ACC (%)	TPR (%)	SPC (%)	PPV (%)	MCC (%)	AUC
*enzymes*	MLP	90.01	100	80.06	83.31	81.67	0.9967
	SVM	89.88	92.31	87.53	88.12	81.77	0.9686
	**DeepLSTM**	**92.92**	**99.31**	**86.57**	**88.04**	**86.75**	**0.9951**
*ion channels*	MLP	87.58	100	75.22	80.07	77.61	0.9972
	SVM	89.36	85.95	92.74	92.23	80.93	0.9613
	**DeepLSTM**	**91.97**	**93.23**	**90.87**	**89.95**	**85.19**	**0.9705**
*GPCRs*	MLP	87.20	76.70	97.77	97.19	77.20	0.9853
	SVM	85.43	86.28	84.60	84.81	74.99	0.9230
	**DeepLSTM**	**91.80**	**83.71**	**100**	**100**	**84.44**	**0.9951**
*nucl. rec*	MLP	88.89	88.24	89.47	88.24	80.19	0.8421
	SVM	85.00	68.90	100	100	72.43	0.9910
	**DeepLSTM**	**91.11**	**95.24**	**87.50**	**86.96**	**83.76**	**0.9206**

From the results summarized in Table 2, The DeepLSTM achieves overall the best prediction results. The accuracies achieved by the DeepLSTM are 92.92% in *enzymes* data set, 91.97% in *ion channels* data set, 91.80% in *GPCRs* data set, 91.1% in *nuclear receptors* data set. and clearly outperform MLP (99.01, 87.58, 87.20, 88.89%, respectively) and SVM (89.88, 89.36, 85.43, 85.00%, respectively). The AUC obtained by the DeepLSTM net are 0.9951 in *enzymes* data set, 0.9705 in *ion channels* data set, 0.9951 in *GPCRs* data set, 0.9206 in *nuclear receptors* data set. However, the MLP net respectively achieve the average AUC of 0.9967, 0.9972, 0.9853 and 0.8421 in four datasets. The SVM respectively achieve the average AUC of 0.9686, 0.9613, 0.9230 and 0.9910 in four datasets. There are five main reasons for the proposed method to produce better results. The first one is that the hierarchical structure of the deep neural network is convert the input data to more complexity space, which is more conducive to complete classification tasks. The second one is that the design of our DeepLSTM not only avoid overfitting effectively, but also makes it possible to train a large number of different neural networks in a short period of time, which makes the network produce better performance. The third one is that the memory units of LSTM can retain more knowledge, which helps to make more accurate decisions at the prediction stage. The fourth is that the LSTM solves the gradient vanishing problem in the Back-Propagation (BP) algorithm, which is helpful to get better prediction model than MLP. The fifth is the use of the validation set helps to train more flexible models.

### Compare with state-of-the-art approaches

In this section, we compared the AUC of our proposed method with that of some state-of-the-art methods including DBSI [10], KBMF2K [37], and NetCBP [38], and the model proposed by Yamanishi et al [7–9] and Wang et al [39]. for the four classes of target-proteins. The results of several methods on four data sets are listed in the Table 4. As it can be observed in Table 4, the AUC of the proposed method is clearly superior in comparison with the AUC of other several methods for the four datasets. The AUC value obtained by our method is 16% higher than those the average in several other methods on *enzymes* dataset. Focus on *nuclear receptors* dataset, the value obtained by our method is 10% higher than those the highest in several other methods, 21% than those the minimum in several other methods. The obviously higher AUC indicates that our scheme obviously outperforms the other compared methods. The results of comparison with other methods also confirm this fact that our method can improve the performance for drug–target interaction prediction. In fact, from the results shown in Table 2, we can see that the other two models (MLP-based and SVM-based) still have higher AUC values than several existing techniques. This shows that our feature extraction strategy can capture the interaction information between drug targets very efficiently and improve the performance of the predictor in predicting the interaction of drug-targets.

**Table 4:** The comparison of the proposed model with seven existing approaches (DBSI, KBMF2K, and NetCBP, and the model proposed by Yamanishi et al and Wang et al.) in terms of the AUC

Datasets	Enzymes	Ion channels	GPCRs	nucl. rec
DBSI	0.8075	0.8029	0.8022	0.7578
NetCBP	0.8251	0.8034	0.8235	0.8394
KBMF2K	0.832	0.799	0.857	0.824
Yamanishi et al.	0.904	0.851	0.899	0.843
	0.892	0.812	0.827	0.835
Wang et al.	0.886	0.893	0.873	0.824
Our method	**0.9951**	**0.9705**	**0.9951**	**0.9206**

## Conclusion

In this paper, we have developed a deep learning-based method to infer potential DTIs using compounds and proteins sequence. To evaluate the ability of our method, we compared it with several state-of-the-art approaches. The experimental results proved that this approach is significantly better than others in terms of performance. Comparing with other classifiers, we have provided initial evidence that DeepLSTM outperforms traditional machine learning system on the DTIs task. For the characterization and quantitative method of drug-target pairs, an interesting scheme was proposed by using SPCA to fuse PubChem fingerprint and protein evolutionary features obtained by the combination of PSSM and LM. Promising results were observed when the characterization method cooperates with three different classifiers, respectively. These results indicate that the proposed scheme has great advantage on feature expression and recognition. We have shown that the proposed method can work well with small dataset, which is distinguish from the predecessor’s methods and goes in its own special way. We also found that prediction quality continues to improve with increasing dataset size. This underscores the value of this model to train and apply very large datasets, and suggests that further performance gains may be had by increasing the data size. On the whole, the theoretical analysis and experimental results give strong theoretical and empirical evidences for the efficacy of using the proposed method to predict DTIs.

## Supplementary information

**Additional file 1: Table S1.** Prediction performance of SVM-based for the *enzymes* datasets in term of ACC, TPR, SPC, PPV, MCC, and AUC. **Table S2.** Prediction performance of SVM-based for the *ion channels* datasets in term of ACC, TPR, SPC, PPV, MCC, and AUC. **Table S3.** Prediction performance of SVM-based for the *GPCRs* datasets in term of ACC, TPR, SPC, PPV, MCC, and AUC. **Table S4.** Prediction performance of SVM-based for the *nuclear receptors* datasets in term of ACC, TPR, SPC, PPV, MCC, and AUC.

## Data Availability

The data and code is available at: https://Deepbiolab.coding.net/s/fbdb894d-2730-425b-bac6-18ba55396bab.

## Abbreviations

ACC: Accuracy
DeepLSTM: Deep long short-term memory
DTIs: Drug-target interactions
LMs: Legendre moments
MCC: Matthew’s correlation coefficient
MLP: Multi-layer perceptron
PPV: Positive predictive value
PSSM: Position specific scoring matrix
RNN: Recurrent neural network
SPC: Specificity
SPCA: Sparse principal component analysis
SVM: Support vector machine
TPR: True positive rate
