# Supplementary material for: A deep learning-based method for drug-target interaction prediction based on long short-term memory neural network
Source: BMC Med Inform Decis Mak. 2020 Mar 18;20(Suppl 2):49. doi: 10.1186/s12911-020-1052-0 (PMC7079345; doi:10.1186/s12911-020-1052-0)
Supplement: Supplementary file 1 — Additional file 1: Table S1. Prediction performance of SVM-based for the enzymes datasets in term of ACC, TPR, SPC, PPV, MCC, and AUC. Table S2. Prediction performance of SVM-based for the ion channels datasets in term of ACC, TPR, SPC, PPV, MCC, and AUC. Table S3. Prediction performance of SVM-based for the GPCRs datasets in term of ACC, TPR, SPC, PPV, MCC, and AUC. Table S4. Prediction performance of SVM-based for the nuclear receptors datasets in term of ACC, TPR, SPC, PPV, MCC, and AUC. [file 12911_2020_1052_MOESM1_ESM.pdf]

Supplementary Material

Table S1. Prediction performance of SVM-based for the *enzymes* datasets in term of ACC, TPR, SPC, PPV, MCC, and AUC.

Model	Testing Set	ACC (%)	TPR (%)	SPC (%)	PPV (%)	MCC (%)	AUC
SVM	1	90.26	97.67	83.50	84.37	82.30	0.9776
	2	90.94	94.28	87.28	89.04	83.41	0.9744
	3	89.57	89.09	90.07	90.31	81.32	0.9621
	4	89.40	91.35	87.50	87.71	81.04	0.9653
	5	89.25	89.18	89.32	89.18	80.81	0.9638
	Average	89.88± 0.7	92.31±3.6	87.53±2.5	88.12± 2.3	81.77± 1.0	0.9686

Table S2. Prediction performance of SVM-based for the *ion channels* datasets in term of ACC, TPR, SPC, PPV, MCC, and AUC.

Model	Testing Set	ACC (%)	TPR (%)	SPC (%)	PPV (%)	MCC (%)	AUC
SVM	1	90.34	88.29	92.44	92.31	82.53	0.9781
	2	88.31	82.65	93.92	93.10	79.21	0.9452
	3	88.81	84.21	93.11	91.95	79.99	0.9440
	4	89.49	87.01	92.20	92.41	81.17	0.9650
	5	89.86	87.59	92.05	91.37	81.75	0.9741
	Average	89.36± 0.8	85.95±2.4	92.74±0.7	92.23± 0.6	80.93± 1.3	0.9613

Table S3. Prediction performance of SVM-based for the *GPCRs* datasets in term of ACC, TPR, SPC, PPV, MCC, and AUC.

Model	Testing Set	ACC (%)	TPR (%)	SPC (%)	PPV (%)	MCC (%)	AUC
SVM	1	85.83	87.68	83.62	86.43	75.45	0.9302
	2	85.04	86.26	83.74	84.96	74.51	0.9232
	3	84.65	84.07	85.11	81.90	73.75	0.9253
	4	86.22	83.33	89.34	89.43	76.21	0.9198
	5	85.43	90.08	81.20	81.34	75.04	0.9166
	Average	85.43± 0.6	86.28±2.7	84.60±2.9	84.81± 3.3	74.99± 0.9	0.9230

Table S4. Prediction performance of SVM-based for the *nuclear receptors* datasets in term of ACC, TPR, SPC, PPV, MCC, and AUC.

Model	Testing Set	ACC (%)	TPR (%)	SPC (%)	PPV (%)	MCC (%)	AUC
SVM	1	86.11	78.26	100	100	75.18	0.9897
	2	86.11	72.22	100	100	75.18	0.9969
	3	83.33	70.00	100	100	71.35	0.9938
	4	86.11	61.54	100	100	71.10	0.9933
	5	83.33	62.50	100	100	69.34	0.9813
	Average	85.00± 1.5	68.90±6.9	100	100	72.43± 2.6	0.9910
